# Liraglutide attenuates hepatic iron levels and ferroptosis in db/db mice

**DOI:** 10.1080/21655979.2022.2051858

**Published:** 2022-03-21

**Authors:** Ji-Xian Song, Ji-Ren An, Qi Chen, Xin-Yue Yang, Cui-Ling Jia, Shan Xu, Ya-shuo Zhao, En-Sheng Ji

**Affiliations:** a Department of Physiology, Institute of Basic Medicine, Hebei University of Chinese Medicine, Shijiazhuang, Hebei, China; b Hebei Technology Innovation Center of TCM Combined Hydrogen Medicine, Shijiazhuang, Hebei, China; c First Clinical College, Liaoning University of Traditional Chinese Medicine, Shenyang, Lioaning, China

**Keywords:** Liver, high glucose, liraglutide, ferroptosis, iron overload, oxidative stress

## Abstract

Liver pathological changes are as high as 21%-78% in diabetic patients, and treatment options are lacking. Liraglutide is a glucagon-like peptide-1 (GLP-1) receptor that is widely used in the clinic and is approved to treat obesity and diabetes. However, the specific protection mechanism needs to be clarified. In the present study, db/db mice were used to simulate Type 2 diabetes mellitus (T2DM), and they were intraperitoneally injected daily with liraglutide (200 μg/kg/d) for 5 weeks. Hepatic function, pathologic changes, oxidative stress, iron levels, and ferroptosis were evaluated. First, liraglutide decreased serum AST and ALT levels, and suppressed liver fibrosis in db/db mice. Second, liraglutide inhibited the ROS production by upregulating SOD, GSH-PX, and GSH activity as well as by downregulating MDA, 4-HNE, and NOX4 expression in db/db mice. Furthermore, liraglutide attenuated iron deposition by decreasing TfR1 expression and increasing FPN1 expression. At the same time, liraglutide decreased ferroptosis by elevating the expression of SLC7A11 and the Nrf2/HO-1/GPX4 signaling pathway in the livers of db/db mice. In addition, liraglutide decreased the high level of labile iron pools (LIPs) and intracellular lipid ROS induced by high glucose in vitro. Therefore, we speculated that liraglutide played a crucial role in reducing iron accumulation, oxidative damage and ferroptosis in db/db mice.

## Graphical Abstract

**Figure:**
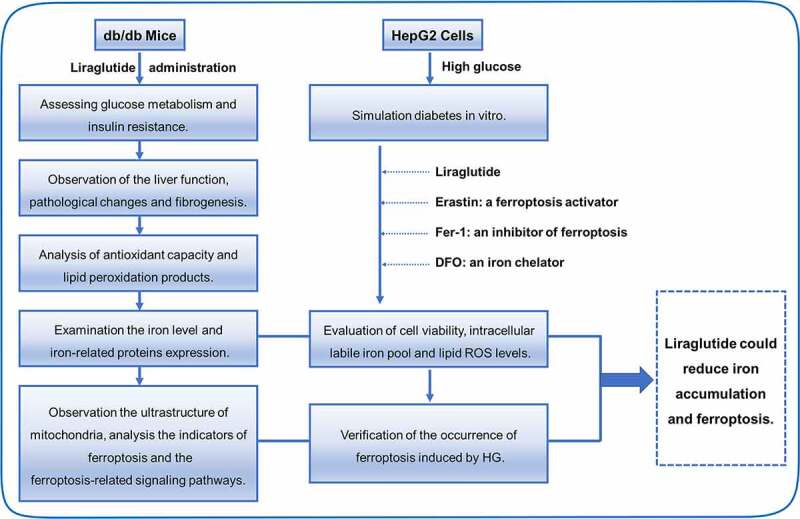

## 1. Introduction

Type 2 diabetes mellitus (T2DM) is one of the most common metabolic disorders characterized by abnormal glucose metabolism lipid metabolism, mainly caused by insulin insufficiency or insulin resistance [1]. Epidemiological studies have shown that the incidence of T2DM is rising rapidly and has become a worldwide public health problem [2]. Long-term high levels of glucose-induced fat and protein metabolism disorders in patients with T2DM may cause severe pathological damage to the central system and peripheral organs, causing relational complications [3,4].

The liver is the most significant metabolic organ responsible for balancing glycolipid metabolism and gluconeogenesis [5]. There is a correlation between diabetes and liver disease, which is inherently complex. Preclinical studies have shown that 21%-78% of diabetic patients present pathological liver changes, such as hepatic steatosis, fatty acid deposition, and fibrosis [6,7]. It has been reported that insulin resistance (IR) and T2DM-induced metabolic syndrome were risk factors for liver damage, such as nonalcoholic fatty liver disease (NAFLD), nonalcoholic steatohepatitis (NASH), and liver fibrosis [8]. Due to the vital link between T2DM and progressive liver disease, in-depth research on the pathogenesis of diabetic liver disease is needed.

To date, research on liver damage in diabetes has mainly focused on oxidative stress in addition to inflammatory and insulin signaling pathways [6,9,10]. Recent studies have found that ferroptosis played an essential role in acute or chronic liver injury [11]. Ferroptosis is a special form of regulated cell death accompanied by iron-dependent lipid peroxides [12]. When excessive iron is accumulated in intracellular, it can generate highly toxic hydroxyl radicals through the Fenton reaction, leading to the biogenesis of polyunsaturated fatty acids (PUFAs), which is the primary startup mechanism of ferroptosis [13,14]. Extensive studies have shown that ferroptosis might aggravate renal tubular damage, endothelial dysfunction, retinal damage, and cognitive impairment in T2DM mice, confirming the correlation between ferroptosis and T2DM [15–18]. Although the role of ferroptosis in diabetic liver injury has rarely been confirmed, it’s necessary to explore this aspect because it promotes fatty liver disease and various complications of T2DM.

Glucagon-like peptide-1 (GLP-1) is an endogenous incretin hormone with glucagon-dependent insulin-stimulating and glucagon-inhibiting effects [19]. Liraglutide is a GLP-1 receptor agonist and has been approved for the clinical treatment of obesity and diabetes [20]. Liraglutide has been demonstrated to be superior to other GLP-1 receptor agonists in blood glucose suppression and overall tolerance [21]. Clinical trials have also indicated its protective effect against NAFLD or NASH [22]. In addition, GLP-1 has also been demonstrated to have an antifibrotic function in organs [23–25]. A recent study has reported that liraglutide had an excellent inhibitory effect on iron overload and ferroptosis in the hippocampus of diabetic mice, providing a good reference for its comprehensive mechanism of action [26].

The protective effect of liraglutide on hepatic ferroptosis of T2DM is still unknown. Our study used db/db mouse and high glucose (HG)-treated HepG2 cells to simulate diabetes and tried to clarify whether ferroptosis was involved in liver injury. In addition, we furtherly evaluated the positive intervention of liraglutide by improving system X_c_^−^ and Nrf2/HO-1/GPX4 signaling pathways. Our findings may provide theoretical support for the treatment of diabetic liver injury.

## 2. Materials and methods

### 2.1 Animals

The db/db mice and non-diabetic littermate db/m mice (male, 8 weeks) were purchased from Changzhou Cavens Experimental Animal Co., Ltd. All the mice were transferred into the experimental animal center and were acclimated for a week. The experimental animal procedures were carried out by the National Institutes of Health Guide for the Care and Use of Laboratory Animals and approved by the Animal Care and Use Committee of Medical Ethics of Hebei University of Chinese Medicine (NO. DWLL2020089).

After one week of adaptive feeding, db/db mice (n = 18) were randomly divided into db/db group (db/db) and liraglutide group (LIRA). The db/m mice were used as the control group (db/m, n = 9). Mice of the LIRA group were given liraglutide (200 μg/kg/d, CSN11311, CSN pharm) by intraperitoneal injection from the first day of 10^th^ to the last day of 14^th^ week. The mice of db/m group received an equal volume of normal saline. Blood glucose level was measured weekly with a blood glucose meter (S59400839789, Sinocare) during the experiment.

### 2.2 Intraperitoneal injection of pyruvate tolerance test

The intraperitoneal pyruvate tolerance test was performed on the 3^rd^ day before sacrificing. After fasting for 14 hours, the mice were intraperitoneally injected with sodium pyruvate (2 g/kg), and blood glucose levels at each time point were measured by tail hemorrhage. Calculate the area under the blood glucose fluctuation curve (AUC).

### 2.3 Pathological staining

H&E staining was used to observe the basic structure of liver tissue. The liver paraffin sections (5 µm) were dewaxed with xylene and rehydrated with gradient alcohol. The sections were stained with hematoxylin, differentiated with hydrochloric acid ethanol, redyed with eosin, dehydrated with gradient alcohol, and vitrificated with xylene. Sirius Red staining is applied to assess liver fibrosis of db/db mice. The liver paraffin sections were dewaxed and rehydration, stained with Sirius Red Stain, dehydrated with ethanol, and vitrificated with xylene. The sections were sealed with mounting medium and collected pictures with a microscope.

### 2.4 Biochemical analysis

The activity of serum alanine aminotransferase (ALT) and aspartate aminotransferase (AST) reflect liver function. The ALT and AST were measured by Biochemical Analytes (iChem-530, Shenzhen Icubio Biotech Co., Ltd.) using the kits according to instructions (Changchun Huili Biotech Co., Ltd.).

### 2.5 Antioxidant and lipid peroxidation products

An appropriate amount of liver tissue was homogenized and centrifuged, and then the supernatant was collected to obtain a homogenate (10% g/V). Protein concentration was measured by the BCA method. The measurement of total superoxide dismutase (SOD) activity, glutathione peroxidase (GSH-Px) enzyme activity, glutathione (GSH), and malonaldehyde (MDA) content were separately carried out according to the kit instruction (NanJing JianCheng Bioengineering Institute). Finally, SOD, GSH, GSH-Px, and MDA levels were measured at 550 nm, 405 nm, 412 nm, and 550 nm using a multifunctional microplate reader. The values were calculated according to the corresponding formulas.

### 2.6 Perls’ staining

Based on our previous method, the Perls’ staining was applied to detect the Fe content and distribution in the liver tissue [27]. After dewaxing and rehydration, the hepatic sections were incubated with 3% H_2_O_2_ at room temperature for 30 min. Then, the sections were soaked into fresh Perls’ dye solution (1%, the mixture of potassium ferrocyanide and hydrochloric acid) for 8 h in the dark. Subsequently, DAB staining and sealed with mounting medium. The mean density of Fe content was calculated by IPP 6.0 software.

### 2.7 Immunohistochemistry

The paraffin sections were dewaxed, rehydrated, and incubated with 3% H_2_O_2_ to remove endogenous peroxidase. Then, sections were added citrate (10 mM, pH 6.0) buffer for antigen repair and sealed with 10% goat serum for 1 h at 37°C. The liver sections were incubated with primary antibodies Transforming Growth Factor-β (TGF-β, Bioss, bs-0086 R) and Acyl-CoA Synthetase Long-Chain Family Member 4 (ACSL4, Affinity, DF12141) for one night at 4°C. Then, the sections were incubated with the HRP-conjugated second antibody for 1 h at 37°C. Finally, sections were stained with a DAB kit and sealed with a mounting medium. The mean density of positive protein was calculated by IPP 6.0 software.

### 2.8 Transmission electron microscope

The transmission electron microscope (TEM) was performed to observe the ultrastructure of mitochondria. After anesthesia, we used a blade to quickly cut and harvest fresh hepatic tissue blocks that were no more than 1 mm^3^. Then, the resin blocks were cut to 60–80 nm thick sections on an ultramicrotome and stained with uranyl acetate and lead nitrate. The images were observed under a HITACHI HT7800 electron microscope.

### 2.9 Cell culture

Human hepatoma HepG2 cells were cultured in RPMI 1640 medium supplemented with 10% fetal bovine serum (Gibco), penicillin, and streptomycin at 37°C with 5% CO_2_. High glucose (HG) cultured conditions were used to simulate diabetes. We cultured the HepG2 cells with different concentrations of HG for 48 h; at the same time, cells were separately treated with liraglutide, Erastin (a ferroptosis activator), Ferrostatin-1 (Fer-1, an inhibitor of ferroptosis), Deferoxamine mesylate (DFO, an iron chelator). At the end of treatment, cell viability or western blot analysis were measured.

### 2.10 Cell viability and related analysis

Cell viability was detected by Cell Counting Kit-8 (CCK-8) kits. HepG2 cells were seeded onto cultured in 96-well plates at 1 × 10^4^ cells per well. After treatment with or without HG, CCK-8 reagents were added to the plates and then incubated at 37°C for 2 h. A multifunctional microplate reader measured the absorbance at 450 nm (Varioskan LUX, Thermo Fisher Scientific).

HepG2 cells were seeded onto 6 cm cell culture dishes at 2 × 10^5^ cells. Calcein-AM (40719ES50, YEASEN Biotechnology) was applied to measure the intracellular labile iron pool (LIP) [28]. After treatment, cells were digested and incubated with 0.25 μM Calcein-AM at 37°C for 15 min. Washing with PBS, the fluorescence intensity (Ex/Em = 490 nm/515 nm) was quantified by a multifunctional microplate reader microplate. The intracellular lipid ROS levels were detected by BODIPY 581/591 C11 probe (D3861, Thermo Fisher Scientific) [29]. Cells were incubated with a 2 μM probe for 37°C for 15 min in the dark. After washed with PBS, the fluorescence spectrum was measured by a multifunctional microplate reader microplate.

### 2.11 Western blot analysis

The liver tissue or cells were prepared to homogenate with cooled PIRA lysate. After centrifugation, the supernatant was collected and the total protein concentration was measured. The 30 μg proteins were separated by SDS-PAGE electrophoresis, and transferred to polyvinylidene fluoride (PVDF) membranes. The blots were incubated with 5% skimmed milk. After washed with TBST, the blots were incubated with primary antibody of Collagen I (Servicebio, GB112543), Collagen III (Servicebio, GB11023), NADPH Oxidase 4 (NOX4, Boster, A00403), 4-Hydroxynonenal (4-HNE, Arigo, ARG23717), Transferrin Receptor 1 (TfR1, Invitrogen, 13–6800), Divalent Metal Transporter 1 (DMT1, Absin, abs112967), Ferroportin 1 (FPN1, Alpha Diagnostic International, MTP11-A), Glutathione Peroxidase 4 (GPX4, Huabio, ET1706-45), the glutamate/cystine antiporter solute carrier family 7 member 11 (SLC7A11, Abclonal, A2413), Nuclear Factor Erythroid 2-Related Factor 2 (Nrf2, Servicebio, GB11962), Heme Oxygenase-1 (HO-1, Servicebio, GB12104), GAPDH (Servicebio, GB15002), α-Tubulin (GeneTex, GTX628802) overnight at 4°C. The second day, blots were incubated with HRP-conjugated secondary antibodies at room temperature. Finally, enhanced chemiluminescent (ECL) method and Image J software were respectively used to detect the immunoreactive protein and analyze the mean gray value.

### 2.12 Statistical analysis

All the data were analyzed by SPSS 23. 0 statistical software, and represented by mean ± SEM. The statistical analysis was measured by one-way ANOVA followed by LSD post hoc test. *p* < 0.05 were considered significant. The graphs were plotted using Prism 8.0 software.

## 3. Results

In this study, we revealed that liraglutide protected against glycometabolism disorders and liver damage in a mouse model of T2DM. Furtherly, liraglutide significantly attenuated oxidative stress damage, iron deposits, and ferroptosis in the liver. Our findings might provide a novelty idea that liraglutide treatment for hepatic abnormalities in T2DM.

### 3.1 Liraglutide alleviates abnormal glucose metabolism in db/db mice

**Figure 1.**
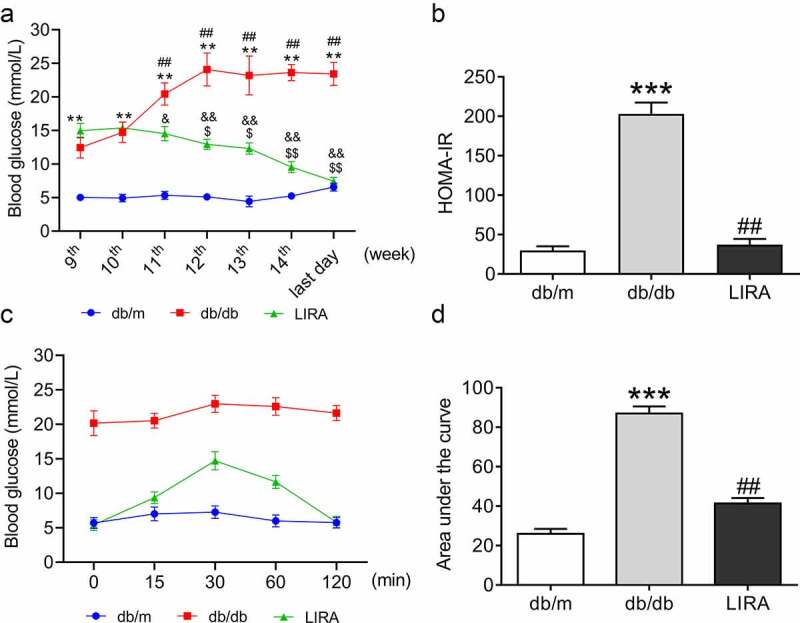
**LIRA improve the disorders of glycometabolism in db/db mice**. (a) The blood glucose of mice from 9^th^ to the last day of 14^th^ week. (The data are shown as the means ± SEM. n = 6. *p < 0.05, **p < 0.01 vs. db/m group. ^##^*p* < 0.01 vs. db/db group 9^th^ week. ^$^*p* < 0.05, ^$$^*p* < 0.01 vs. LIRA group 9^th^ week. ^&^*p* < 0.05, ^&&^*p* < 0.01 vs. db/db group.). (b) The HOMA-insulin resistance (HOMA-IR) of db/m, db/db and LIRA groups. (c) The intraperitoneal glucose tolerance test of mice with 120 min. (d) The area under of the curve as shown in panel C. The data are shown as the means ± SEM. n = 6. ***p < 0.001 vs. db/m group. ^##^*p* < 0.01 vs. db/db group.

We used db/db mouse, a widely used rodent model, to study T2DM and related complications [30]. At the beginning of the 9^th^ week, we measured blood glucose in all groups. The mice were then administered liraglutide for 5 weeks. Blood glucose measurement continued during the liraglutide treatment period. Compared to db/m mice, db/db mice exhibited significantly higher levels of fasting blood glucose (Figure 1(a), *p* < 0.01). After liraglutide intervention, the elevated blood glucose in db/db mice was significantly decreased (Figure 1(a), *p* < 0.05 and *p* < 0.01). The data revealed that the db/db mice showed considerably increased HOMA-IR at the last day of 14^th^ week, and the values declined when administered liraglutide (Figure 1(b)). At the same time, we evaluated intraperitoneal pyruvate tolerance, which reflects gluconeogenesis. Compared to db/m mice, the values of intraperitoneal pyruvate tolerance significantly elevated within 120 min; and these values decreased during treatment with liraglutide (Figure 1(c-d)). Based on the above results, we confirmed that liraglutide significantly improves glycometabolism and IR in db/db mice.

### 3.2 Liraglutide significantly attenuates liver damage in db/db mice

**Figure 2.**
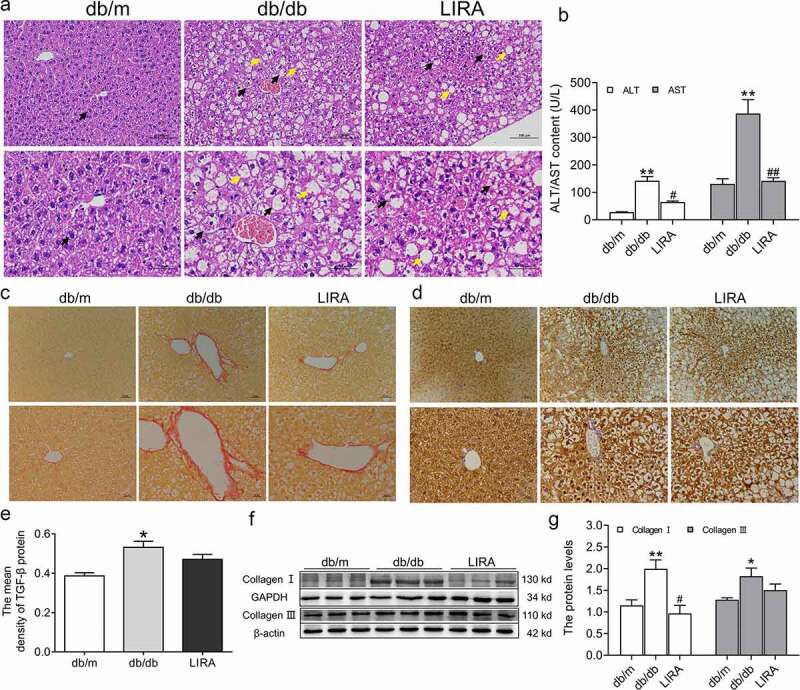
**The liver dysfunction and fibrosis in db/db mice**. (a) The liver H&E staining of db/m, db/db and LIRA groups (n = 3). The black arrow represents the normal hepatocytes (db/m group) or ballooning hepatocytes (db/db and LIRA groups). The yellow arrow represents the adipocytes. (b) The serum ALT and AST content (n = 4–5). (c) Sirius red staining of mice liver (n = 3). (d) The immunohistochemical staining of TGF-β protein (n = 3). (e) The mean density of TGF-β protein as shown in panel D. (f-g) The expression and statistics of Collagen I and Collagen III proteins in liver tissue (n = 6). The results are presented as the mean ± SEM. *p < 0 05, **p < 0 01 vs. db/m group. ^#^*p* < 0 05, ^##^*p* < 0 01 vs. db/db group.

H&E staining was used to assess the pathological structure in the liver. There were no visible architectural alterations in the hepatic sections from db/m mice. In the db/db mice sections, the following structural alterations were observed: the hepatic lobule structure was clear, the hepatic cell cord arrangement was disordered, numerous hepatocytes were balloon-like with vacuolated cytoplasm, and some circular lipid vacuoles were present (Figure 2(a)). Treatment with liraglutide improved the symptoms induced in db/db mice. To gain further insight into the effect of liraglutide on liver impairment, we measured the levels of ALT and AST in serum. The increased ALT and AST contents decreased after administration of liraglutide in db/db mice (Figure 2(b)).

We also assessed liver fibrogenesis by Sirius Red staining and found that the fibrotic areas were significantly expanded in the db/db mice (Figure 2(c)). Immunohistochemical staining further indicated that TGF-β increased in the livers of db/db mice (Figure 2(d-e)). Furthermore, western blot analysis showed that collagen I and collagen III increased in the liver tissue of the db/db group (figure 2(f-g)). Together, these results demonstrated that the db/db mice have a liver injury with fibrosis. After liraglutide treatment, the fibrotic areas were decreased (Figure 2(c)), and the protein levels of collagen I were lower (figure 2(f-g)). Moreover, the TGF-β and collagen III protein levels decreased, but there were no significant differences between the db/db and LIRA groups (Figure 2(d-g)). These results revealed that treatment with liraglutide recovers the injured fibrotic liver.

### 3.3 Liraglutide markedly improves oxidative stress damage in db/db mice

**Figure 3.**
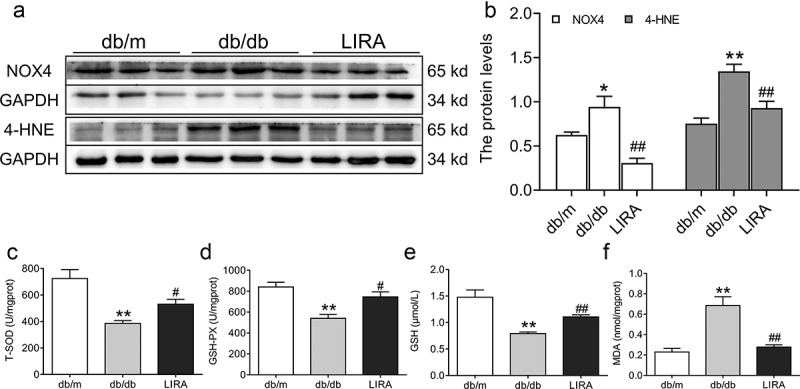
**The oxidative stress levels in the liver tissue of db/db mice**. (a-b) The expression and statistics of NOX4 and 4-HNE in liver tissue (n = 6). (c-d) The total SOD and GSH-PX activity (n = 5–6). (e-f) The GSH and MDA content (n = 5–6). The results are presented as the mean ± SEM. *p < 0 05, **p < 0 01 vs. db/m group. ^#^*p* < 0 05, ^##^*p* < 0 01 vs. db/db group.

Oxidative stress was measured by estimating antioxidant capacity and lipid peroxidation. The NOX4 (*p* < 0.05) and 4-HNE (*p* < 0.01) protein levels significantly increased in the liver tissue of db/db mice (Figure 3(a-b)), but these levels significantly decreased after treatment with liraglutide. We also found that the total SOD and GSH-PX activities and GSH content were lower in the db/db group than in the db/m group (*p* < 0.01, Figure 3(c-e)). However, the downward trend was reversed after treatment with liraglutide (*p* < 0.05 and *p* < 0.01). MDA content was significantly elevated in the liver tissue of db/db mice (*p* < 0.01), and liraglutide markedly decreased the MDA levels (*p* < 0.01, figure 3(f)).

### 3.4 Liraglutide weakens iron deposits in the livers of db/db mice

**Figure 4.**
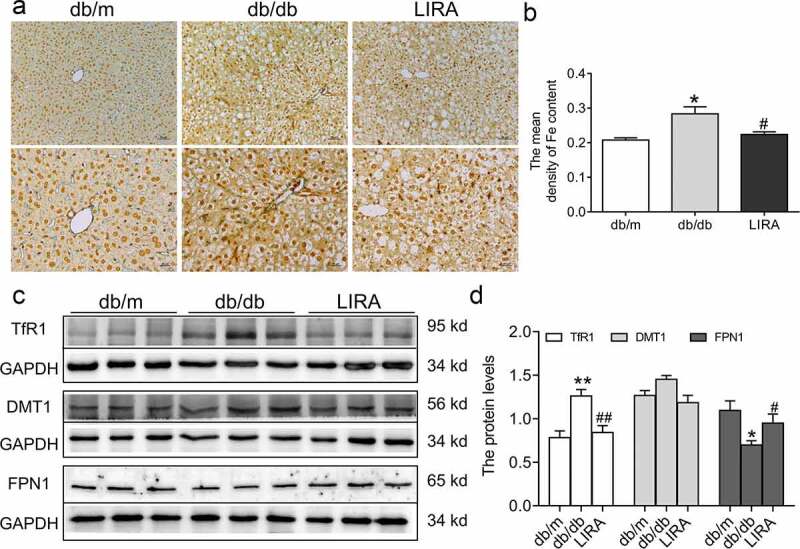
**The iron levels and iron-related transportproteins in the liver tissue of db/db mice**. (a) The Perls’ staining of liver tissue (n = 3). (b) The mean density of Fe content as shown in panel A. (c-d) The expression and statistics of TfR1, DMT1 and FPN1 proteins (n = 6). The results are presented as the mean ± SEM. *p < 0 05, **p < 0 01 vs. db/m group. ^#^*p* < 0 05, ^##^*p* < 0 01 vs. db/db group.

Because excess iron aggravated ROS generation and lipid peroxidation damage, we detected iron levels in the livers of db/db mice. Compared to the db/m group, Perls’ staining revealed that the iron content was significantly elevated (*p* < 0.05) in the liver, indicating iron overload in db/db mice (Figure 4(a-b)). Next, we explored the underlying mechanism by which regulatory proteins regulate iron intake and release. Western blot analysis confirmed the higher level of TfR1 (*p* < 0.01) and lower level of FPN1 (*p* < 0.05) in the liver tissue of db/db mice (Figure 4(c-d)). The expression of DMT1 showed a slightly increasing trend, but there was no statistically significant difference (Figure 4(c-d)). Liraglutide treatment significantly reduced the mean density of iron content in hepatic sections by down-regulating TfR1 protein expression and up-regulating FPN1 protein expression (Figure 4(a-d)). These observations suggested that db/db mice exhibit iron overload and iron metabolic disorder, attributed to ROS generation, which LIRA could rescue.

### 3.5 Liraglutide prevents ferroptosis in the livers of db/db mice

**Figure 5.**
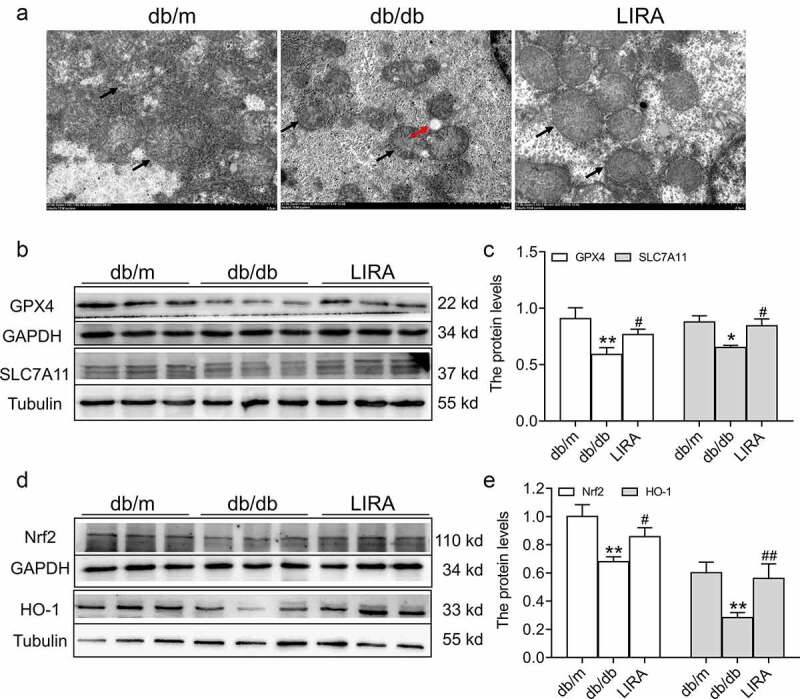
**The ferroptosis related signaling pathway in the liver tissue of db/db mice**. (a) The ultrastructure of mitochondria observed by TEM (n = 3). The black arrow represents the mitochondrial outer membrane. The red arrow represents regional focal fatty infiltration. (b-c) The expression and statistics of GPX4 and SLC7A11 proteins (n = 6). (d-e) The expression and statistics of Nrf2 and HO-1 proteins (n = 6). The results are presented as the mean ± SEM. *p < 0 05, **p < 0 01 vs. db/m group. ^#^*p* < 0 05, ^##^*p* < 0 01 vs. db/db group.

First, we observed the ultrastructure of mitochondria. Compared to the db/m group, the mitochondria were smaller with the fragmentation of ridges and regional focal fatty infiltration in db/db mice (Figure 5 (a)). After treatment with liraglutide, the mitochondrial ultrastructure was improved. We next evaluated the expression of GPX4, the classic marker of ferroptosis. As shown in Figure 5(b-c), Western blot analysis showed that the GPX4 levels were significantly declined in the db/db group (*p* < 0.01). In addition, SLC7A11 protein levels were also decreased in the db/db group (*p* < 0.05). However, treatment with liraglutide elevated the expression of GPX4 and SLC7A11 (*p* < 0.05, Figure 5(b-c)). We also found that the expression of the antioxidant signaling factors, Nrf2 and HO-1, was decreased in the liver tissue of db/db mice (*p* < 0.01, Figure 5(d-e)). Liraglutide significantly increased the levels of Nrf2 and HO-1 in db/db mice (*p* < 0.05 and *p* < 0.01, Figure 5(d-e)). These results suggested that liraglutide attenuates ferroptosis in the livers of db/db mice.

### 3.6 Liraglutide inhibits the HG-induced ferroptosis of HepG2 cells

**Figure 6.**
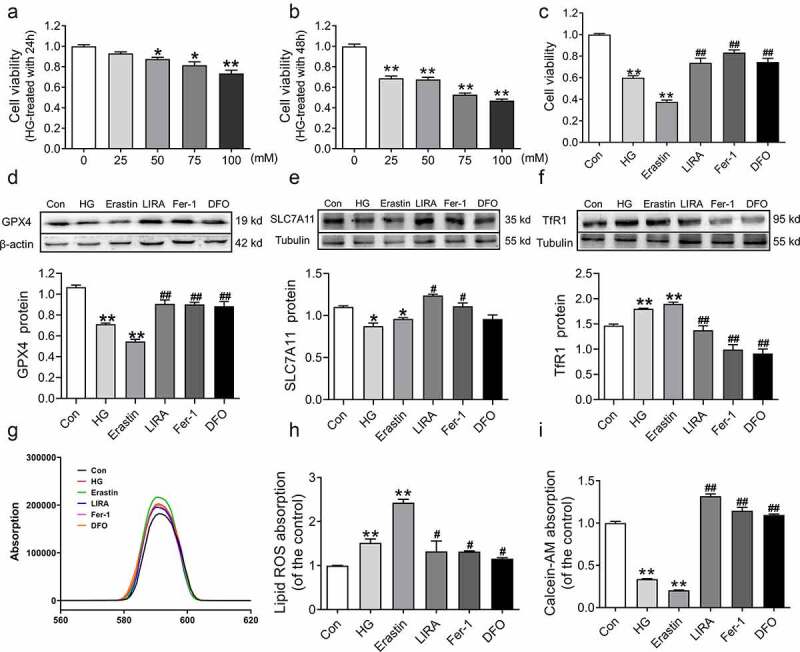
**Liraglutide inhibited the ferroptosis in HepG2 treated with high glucose**. (a-b) The cell viability of HepG2 cells treated with high glucose with 0, 25, 50, 75, 100 mM for 24 h and 48 h (n = 6, *p < 0 05, **p < 0 01 vs. Con group). (c) The cell viability of HepG2 cells treated high glucose (HG, 75 mM), Erastin (200 nM), liraglutide (LIRA, 100 nM), Fer-1 (2 μM), DFO (200 μM). (d-f) The expression and statistics of GPX4, SLC7A11 and TfR1 proteins (n = 3). (g) The fluorescence absorption spectrum of BODIPY 581/591. (h) The folds changes of fluorescence absorption as shown in panel G. (i) The folds changes of fluorescence absorption of Calcein-AM with different groups (n = 6). The results are presented as the mean ± SEM. *p < 0 05, **p < 0 01 vs. Con group. ^#^*p* < 0 05, ^##^*p* < 0 01 vs. HG group.

To further verify whether liraglutide suppresses ferroptosis in db/db mice, we cultured HepG2 cells with HG to simulate hyperglycemia *in vitro*. We used different concentrations of HG treatment for 24 h and 48 h (Figure 6(a-b)), and we selected a final concentration of 75 mM HG for 48 h, in which cell viability significantly declined (*p* < 0.05, Figure 6(b-c)). Meanwhile, we used Erastin (200 nM), liraglutide (100 nM), Fer-1 (2 μM), and DFO (200 μM) during HG treatment. Compared to the control group, the cell viabilities significantly decreased in the HG (*p* < 0.01) and Erastin (*p* < 0.01) groups (Figure 6(c)). Moreover, the cell viabilities were recovered in the HG+LIRA group, similar to the effect observed in the HG+Fer-1 and HG+DFO groups (Figure 6(c)). Western blot analysis revealed lower levels of GPX4 and SLC7A11 as well as higher levels of TfR1 in the HG group (Figure 6(d-f)), which was consistent with the results *in vivo*. However, the reverse results were obtained in the HG+LIRA group, HG+Fer-1 and HG+DFO groups (Figure 6(d-f)). In addition, the MFI of BODIPY 581/591 C11 was elevated in the HG group (Figure 6(g-h)). The lower Calcein-AM MFI in the HG group suggested that more LIP needed to be added to the treatment (Figure 6(i)). Liraglutide reversed the changes in the MFI of Calcein-AM and BODIPY 581/591 C11 in HG-treated HepG2 cells (Figure 6(g-i)). These results indicated that high glucose induces ferroptosis and that liraglutide suppresses ferroptosis.

## 4. Discussion

The pathophysiological mechanism of T2DM involves many different tissues, but the liver and T2DM are the most closely related. Increased gluconeogenesis in the liver is a hallmark of T2DM [31]. Under normal physiological conditions, the liver can quickly adapt to the metabolic transition between eating and fasting as well as respond to postprandial insulin secretion to inhibit hepatic gluconeogenesis, thereby limiting glucose production [32]. Study has shown that liver damage may lead to increased IR and abnormal gluconeogenesis; in addition, disorders of glucose and lipid metabolism further aggravate the process of oxidative stress and inflammation in liver cells [33]. Study has also found that in patients with T2DM, the main source of endogenous glucose production was gluconeogenesis rather than glycogenolysis, emphasizing that maintaining a normal rate of gluconeogenesis was the key to avoiding the occurrence of T2DM [34]. Our results showed that db/db mice had significantly increased glucose and abnormal gluconeogenesis, and they showed that liraglutide effectively improved these processes, which agreed with previous reports [35]. Based on these results, we focused on liver changes.

Fatty degeneration and fibrotic lesions of the liver caused by T2DM have been widely confirmed, and the extent depends on the process of T2DM [36,37]. In T2DM, IR leads to liver steatosis and NASH, promoting the progression of liver fibrosis [38]. Recent studies have demonstrated that hyperglycemia plays an essential role in promoting the activation and proliferation of hepatic stellate cells (HSCs) as well as collagen formation [39,40]. When the liver injury occurs, HSCs are activated and transformed, and they produce TGF-β, which promotes inflammation, migration, proliferation, and fibrogenesis. In particular, it starts HSCs synthesize type I collagen in large quantities [41,42]. Liraglutide has been demonstrated to reverse cholesterol transport, improve lipid metabolism, and accumulate liver lipid in high-fiber diet-fed db/db mice [43]. There is also clinical evidence that liraglutide reduces intrahepatic vascular resistance in patients with liver cirrhosis; and significantly improves fibrosis, the HSC phenotype, and endothelial function [44]. Our results showed that the elevated levels of ALT and AST in db/db mice represent a significant deficit in liver function. In addition, fibrosis was found in the liver of db/db mice, accompanied by high levels of TGF-β and collagen I. Moreover, liraglutide significantly improved the abovementioned changes, further suppressing fatty degeneration and fibrosis of the liver.

Oxidative stress plays an essential role in the process of diabetes [45]. The abnormally high levels of free radicals and the simultaneous decline of antioxidant defense mechanisms cause damage to organelles and enzymes, increase lipid peroxidation, promote the development of IR, and promote the formation of diabetic complications [46]. Unsaturated fatty acids on mitochondrial membranes are prime targets of ROS attack when oxidative stress occurs, leading to lipid peroxidation and reactive lipid production. NADPH oxidases (NOXs) are critical enzymes of REDOX signaling specifically expressed in mitochondria; NOXs catalyze the reduction of oxygen molecules to form ROS, such as superoxide or peroxide [47]. MDA and 4-HNE are the two main end products of lipid peroxidation, and their content changes indirectly reflect the degree of tissue peroxidation damage [48]. As an antioxidant metalloenzyme, SOD catalyzes the disproportionation of superoxide anion radicals to generate oxygen and hydrogen peroxide, which plays an essential role in the fight against oxidative stress [49]. A previous study has confirmed that HG exposure significantly increased the ROS content in mouse liver [50]. Our data showed that SOD and NOX4 were respectively decreased and increased, and 4-HNE and MDA were all overexpressed, indicating that db/db mice are unable to combat oxidative stress and lipid peroxidation. Liraglutide significantly improved the level of antioxidative stress in the liver of diabetic mice, which may be one of the important mechanisms by which liraglutide improves diabetic liver damage.

**Figure 7.**
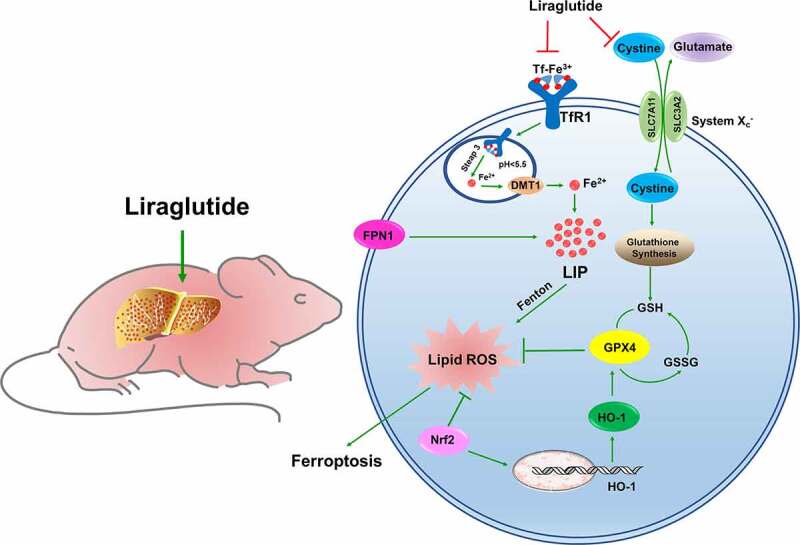
**The schematic description of ferroptosis involved in liver of db/db mice**. The diabetic mice owned a high glycemic index in whole body, accompanied with hepatic fibrosis and iron overload. The high level of TfR1 and lower level of FPN1 contributed to elevated LIP in liver. The excessive Fe^2+^ could aggravate lipid ROS generation by Fenton action. The unbalance system X_c_^−^ could lead to glutathione exchange was inhibited, GSH synthesis was decreased, GPX4 was declined, resulting in ferroptosis. Nrf2 could promote HO-1 expression to elevated GPX4, against as lipid ROS injury. The liraglutide could decrease the overload iron level in liver by adjusting the TfR1 and FPN1 expression. The liraglutide could improve system X_c_^−^ and Nrf2 /HO-1 pathway to against to ferroptosis in liver of db/db mice.

A previous study has reported that iron levels play a role as a ‘second hit’ observed in T2DM patients [51] and db/db mice [27]. The iron contributes to the ROS pool through the Fenton reaction [52]. Moreover, various iron-containing enzymes, such as NOXs, are involved in the process of ferroptosis [53]. In the liver, many iron ions are stored in HSCs, and iron overload may affect the development of liver fibrosis by aggravating lipid peroxidation [54]. Studies have shown that excess serum and hepatic iron in db/db mice trigger oxidative stress via Fenton chemistry [26,30]. Transferrin (Tf) binds with Fe^3+^ and is the main form of iron transport in the blood into cells. Tf-Fe^3+^ binds to TfR1, and the complex then enters the cell via endocytic vesicles. When the pH value of the vesicle changes, Fe^3+^ will be released and reduced to Fe^2+^, and then transported to the cytoplasm through DMT1 [55]. In normal physiological conditions, the LIP is maintained at a very low level. However, under high levels of oxidative stress, superoxide could induce Fe2+ release from [4Fe–4S] cluster, heme, and ferritin, eventually causing iron toxicity [52,56]. In the present study, the db/db mice had high TfR1 levels and low FPN1 levels, leading to iron deposition in the liver (Figure 7). In high-glucose milieu, glucose could be oxidized by transition metal (iron) enediol radical anion and simultaneously converted into ketoaldehydes and superoxide anion radicals, which disproportionate superoxide anion to hydrogen peroxide. Further, if in a state of iron overload, highly toxic hydroxyl radicals are generated [46]. However, liraglutide reversed iron overload in the liver, which may indirectly alleviate the accumulation of peroxides and lipid peroxides.

Similarly, ferroptosis is a cell death method mediated by iron-dependent lipid peroxidation [12,57]. The essence of ferroptosis is the depletion of GSH and the decrease in GPX4 activity [58]. The cystine-glutamate antiporter system (System Xc^−^) is an amino acid antiporter that consists of the light chain subunit, SLC7A11, and the heavy chain subunit, SLC3A2 (Figure 7) [59]. System Xc^−^ mainly mediates the exchange of cystine and glutamate, thus promoting the synthesis and stabilization of intracellular GSH [12]. GSH reduces toxic lipid oxides to nontoxic alcohols, which plays a vital role in scavenging free radicals and protecting cells from oxidative stress losses [60]. The center of GPX4 is selenocysteine, which catalyzes the transformation of reduced GSH into oxidized GSH [61]. When System Xc^−^ is blocked, glutathione cannot be exchanged, resulting in intracellular glutamate accumulation, decreased GSH synthesis and GPX4 activity, resulting in cell ferroptosis (Figure 7) [62]. Our results revealed lower GSH and GPX4 in db/db mice, suggesting an imbalance in System X_c_^−^ in the livers of diabetic mice. Study has reported that silencing of SLC7A11 was rendered HT-1080 cells more sensitive to ferroptosis [63]. This is consistent with our results that the inactivation of SLC7A11 leads to the breakdown of System Xc^−^ stability. We further verified the occurrence of hepatocyte ferroptosis and the effect of liraglutide on db/db mice and high glycemic cell models, which broadened the mechanism and treatment strategies of diabetic liver injury.

Nrf2 is an important transcription factor that regulates cellular antioxidant stress. Under oxidative stress, ROS leads to Keap1 inactivation and Nrf2 phosphorylation; phosphorylated Nrf2 is transferred to the nucleus, binds to antioxidant response elements and induces the expression of the downstream antioxidant gene, HO-1 [64,65]. Recent studies have confirmed that the Nrf2 target plays a crucial role in mediating iron/heme metabolism, including regulating iron homeostasis in unstable iron pools [66]. It is worth noting that many proteins and enzymes responsible for preventing lipid peroxidation and causing ferroptosis are Nrf2 target genes, thus illustrating the positive effect of Nrf2 on inhibiting ferroptosis (Figure 7) [67]. Several drugs can upregulate the expression of the hepatic Nrf2/HO-1 signaling pathway to improve liver fibrosis or injury [68]. Liraglutide has been demonstrated to protect neuronal cells in the brain of diabetic rats by activating the Nrf2/HO-1 signaling pathway [69], which was consistent with the results of liraglutide activating the Nrf2/HO-1 pathway in the livers of diabetic mice in the present study. These results show that liraglutide prevents the occurrence of oxidative stress, iron overload and ferroptosis by activating Nrf2/HO-1, thereby improving liver fibrosis.

Taken together, these findings suggested that ferroptosis is involved in hepatic fibrosis and leads to liver injury in db/db mice, which may be attributed to increased ROS and lipid peroxidation as well as iron deposition. Interestingly, we found that ferroptosis in hepatocytes is suppressed by the Nrf2/HO-1 pathway. These preliminary findings and related hypotheses need further confirmation and will be the basis of future research on mechanisms involved in liraglutide therapy for hepatic fibrosis injury in clinical settings.

## 5. Conclusion

In conclusion, the present study demonstrated that liraglutide improves diabetes-induced hepatic fibrosis, which may be related to the inhibition of the ferroptosis pathway. In addition, the reduction of oxidative stress, lipid peroxidation and the alleviation of iron overload by liraglutide are important factors in its ability to inhibit ferroptosis. Thus, diabetic liver injury and the clinical application of liraglutide should be further studied.

## Highlights

- Ferroptosis was activated in liver of db/db mice.
- Liraglutide attenuated iron overload and ferroptosis in liver of db/db mice.
- Liraglutide improved System Xc^−^ and Nrf2/HO-1 signaling pathway during the process.
